# Nomenclature for Human Infections Caused by Relapsing Fever *Borrelia*

**DOI:** 10.3201/eid2905.230195

**Published:** 2023-05

**Authors:** Paul S. Mead

**Affiliations:** Centers for Disease Control and Prevention, Fort Collins, Colorado, USA

**Keywords:** *Borrelia*, *Borrelia miyamotoi*, relapsing fever, bacteria, tickborne, vector-borne infections

**To the Editor:** Vazquez et al. report a convincing case of relapsing fever caused by *Borrelia lonestari* bacteria (*1*). This discovery highlights an existing problem with the nomenclature for relapsing fever. 

Tick-borne relapsing fever (TBRF) is the name given to illness caused by several genospecies of relapsing fever *Borrelia* bacteria, all of which are transmitted by argasid (soft) ticks (*2*). The limitations of this term became apparent after discovery of *B. miyamotoi*, a related genospecies that is transmitted by ixodid (hard) ticks and causes illness that differs epidemiologically from traditional TBRF (*3*). Consequently, 3 terms are used in the scientific literature to describe *B. miyamotoi* infections: *Borrelia miyamotoi* disease, hard tick–borne relapsing fever, and hard tick relapsing fever (*3*,*4*). In the interest of standard nomenclature, it is worth considering objectively the relative merits of each term.

The term *Borrelia miyamotoi* disease (BMD) is problematic because it is species specific and cannot accommodate the discovery of related pathogens transmitted by ixodid ticks, including potentially *B. lonestari* (*1*,*3*). Disease names are most serviceable as umbrella terms that exist above the species level (e.g., Lyme disease, shigellosis).

The term hard tick–borne relapsing fever has a different problem, a grammatical one. The word hard rightly modifies tick, not tick-borne, which is a mode of transmission. This problem is solved by shortening to hard tick relapsing fever. The suffix “-borne” is not essential for clarity, as demonstrated by other established names (e.g., sand fly fever, Colorado tick fever) (*2*).

In the absence of a formal nomenclature decision by the World Health Organization, the following terms are consistent with precedent, epidemiologically useful, linguistically sensible replacements for TBRF: hard tick relapsing fever (HTRF) for illness caused by relapsing fever–clade *Borrelia* transmitted by ixodid ticks, and its congener, soft tick relapsing fever (STRF), for related agents transmitted by argasid ticks.
